# Intraoperative hydrostatic reduction of intussusception

**DOI:** 10.4103/0971-9261.43807

**Published:** 2008

**Authors:** Uday Sankar Chatterjee, Ajoy Ghosh, Ashoke Kumar Basu, Partha Pratik Mukhopadhyay

**Affiliations:** Department of Pediatric Surgery, Park Children’s Centre for Treatment and Research, 4 Gorky Terrace, Kolkata - 700 017, India

**Keywords:** Fluoroscopy, hydrostatic reduction, intussusception, primary laparotomy, ultrasonography

## Abstract

**Aims:**

To find out an easier way of reduction of intussusception during open surgery to avoid unnecessary bowel injury.

**Materials and Methods:**

Under general anesthesia, before laparotomy, warm normal saline was infused into the rectum with a Foley catheter and an intravenous drip set maintaining the level of the bottle at 80 cm above the operating table. After opening the abdomen, pressure was applied on the colon filled with normal saline distal to the intussusceptum. The pressure was transmitted to the intussusceptum and the walls of the intussuscipient and caused reduction of intussusception without any injury to the intussuscipient and intussusceptum. This procedure was performed on those patients on whom laparotomy was performed as a primary procedure due to nonavailability of fluoroscopy or ultrasonography.

**Results:**

Between August 1998 and July 2005, we had six patients of mean (range) age 11 months (7–17 months). In two cases, at laparotomy, the intussusceptions were found to have already reduced.

**Conclusions:**

Gentle finger pressure is necessary for reduction of intussusception. This subjective “gentleness” is dependant on experience of the surgeon and varies from person to person. Focal pressure on the intussuscipient and apex of the intussusceptum by the finger during reduction may be more damaging than the diffusely transmitted hydrostatic pressure even by a less-experienced surgeon. This will avoid the needless resection and anastomosis of the intestine on many occasions.

## INTRODUCTION

An intussusception is a surgical emergency, and delayed presentation is common in our country. Delay in treatment is a significant independent risk factor for increased morbidity and mortality.[1] Some patients with complications require a primary laparotomy. Standard barium and air reductions for intussusceptions are successful under these conditions in only 13–22% of the cases.[2]

For logistic reasons, ultrasonographic and fluoroscopic cover were not available at certain times (on weekends, holidays, at night, etc.). In these situations, we had to perform primary laparotomy in five patients over a period of 7 years in a well-equipped institution and we tried hydrostatic reduction peroperatively.

According to Daniel,[3] “The intussusception may be reduced by ‘gentle finger pressure’ on the apex of the intussuscepted intestine.…” In addition, “With ‘gentle continuous pressure’ on the apex of the intussusceptum, gradual reduction of the intestine is usually possible.” Still, gut injury during reduction is not an uncommon problem, particularly by the less-experienced surgeons.

The present study, with peroperative hydrostatic pressure, was performed to find out whether atraumatic reduction of gut in the intussusception is possible or not.

## MATERIALS AND METHODS

We had the following inclusion criteria to perform this study: (1) patients with clinically confirmed intussusceptions, (2) delay in diagnosis of more than 48 h and (3) nonavailability of fluoroscopy and ultrasonography. From August 1998 to July 2005, we had six patients of mean (range) age of 11 months (7–17 months). One of the six patients was excluded from the study for a reason discussed below.

Patients were resuscitated with intravenous (IV) fluids, nasogastric suction and antibiotics. All patients received two or three enemas before anesthesia. We consider this an important step. This cleans the distal bowel of fecal matter and prevents fecal soiling of the peritoneum in case of an accidental perforation. One patient had complete reduction of intussusception with this preoperative enema alone and was excluded from the report.

The presence of a lump was reconfirmed by examination under anesthesia and a Foley catheter of the largest appropriate size (14–16F) for the patient was inserted into the rectum and the balloon was inflated. Normal saline warmed to body temperature was infused into the Foley catheter with an IV drip set, maintaining the saline bottle level at 80 cm above the operating table. Distenstion of the left flank and extension towards the right was visible.

**Figure 1:**
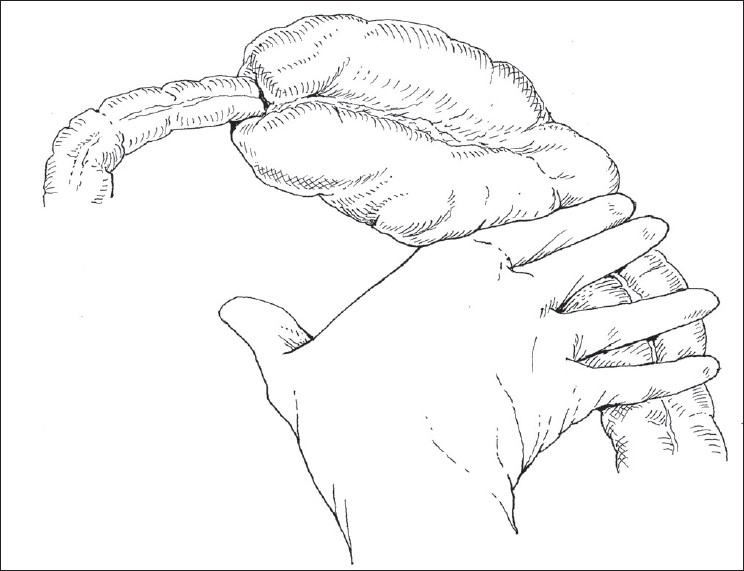
Pressure applied on the colon filled with normal saline, distal to the intussusceptum

Laparotomies were performed through small right transverse incisions immediately above the umbilicus. On two occasions, at laparotomies, the intussusceptions were found reduced and only edematous and ecchymosed gut was seen. On three occasions, we applied pressure on the saline-filled intussuscipient, distal to the intussusceptum without exteriorizing the lump [Figure 1]. This pressure was transmitted to the intussusceptum and the walls of the intussuscipient and caused reduction without any serosal tear or other injury to the intussuscipient and intussusceptum. No pathological lead point was found in any of these five cases.

## RESULTS

In no patient was resection of gut necessary. All patients had uneventful recovery. During the follow-up period [Mean (range) 46 (1–84) months], there was no recurrence.

## DISCUSSION

Initial diagnosis may be carried out by contrast enemas controlled by fluoroscopy or by ultrasonography.[4–6] Management by nonsurgical means with imaging is the preferred method of treatment. Ten to 20% of the patients need surgical treatment. Reduction of intussusception demands patience and gentle finger pressure. “Gentleness” of finger pressure is dependant on the experience of the surgeon, varying from person to person. It is difficult to teach this “gentleness” and probably it comes from experience. However, monitoring of transmitted digital pressure on intussuscipient is possible through the level of the saline column.

From our experience, focal pressure on the intussuscipient and the apex of the intussusceptum may be more damaging than a diffusely transmitted hydrostatic pressure by this procedure delivered by a less-experienced surgeon. This procedure seems to be helpful in reducing the rate of resection and anastomosis, the incidence of which at times maybe as high as 25%.[7]

This type of peroperative hydrostatic reduction may be helpful for the beginners and surgeons who do not come across the condition frequently and may also be suggested during laparoscopy.[8]

This study was performed on a small number of patients. Further studies are necessary to establish its superiority over standard reduction of intussusception by gentle finger pressure.
